# Mimi-PAGE, an open-source implementation of the PAGE09 integrated assessment model

**DOI:** 10.1038/sdata.2018.187

**Published:** 2018-09-25

**Authors:** Frances C. Moore, James Rising, Niklas Lollo, Cecilia Springer, Valeri Vasquez, Alex Dolginow, Chris Hope, David Anthoff

**Affiliations:** 1 Environmental Science and Policy, University of California, Davis, CA, 95616, USA; 2 Grantham Research Institute on Climate Change and the Environment, London School of Economics, London, WC2A 2AE, UK; 3 Energy and Resources Group, University of California, Berkeley, Berkeley, CA, 94720, USA; 4 Cambridge Judge Business School, University of Cambridge, Cambridge, CB2 1AG, UK

**Keywords:** Climate-change policy, Climate and Earth system modelling, Environmental economics

## Abstract

Integrated Assessment Models (IAMs) have become critical tools for assessing the costs and benefits of policies to reduce greenhouse gas emissions. Three models currently inform the social cost of carbon dioxide (SCCO_2_, the net present value of damages from one additional ton of CO_2_) used by the US federal government, several states, and Canada. Here we present a new open-source implementation of one of these models (PAGE09) in the Julia programming language using a modular modeling framework (Mimi). Mimi-PAGE was coded using best coding practices (such as multiple code reviews by different individuals during development, automated testing of newly-committed code, and provision of documentation and usage notes) and is publicly available in a GitHub repository for community inspection and use under an open source license. In this paper we describe the Julia implementation of PAGE09, show that output from Mimi-PAGE matches that of the original model, and perform comparisons of the run time between the two implementations.

## Introduction

### Open-Source IAMs

IAMs model the global economy, climate system, and connections between them via greenhouse gas emissions and climate damages. They are useful tools for policymakers as well as scientists and economists seeking to understand the relationship between energy policy and the costs of climate change-induced damages. Federal regulators in the United States use estimates of the SCCO_2_ informed by three IAMs, as does Canada and several US states^1–4^. However, to maximize their usefulness, the IAMs that support both policy recommendations and academic research could be made more accessible and available for each community’s use^5^. Several key standards can enable sound, reproducible, and communicable science using IAMs: open-source code, a platform for model inter-operability, consistent programming conventions, reduced computational processing time, automated testing for new commits to the repository, and documented methodology^6^.

Our endeavor meets these standards first by translating a model (PAGE09)^7^, which formerly relied on proprietary software (specifically Microsoft Excel and the @RISK Excel plug-in), into an open-source and high-performance computing language, Julia, which follows much of the syntax of the widely-used MatLab language. Second, our model version uses a component-based modelling framework (Mimi) that allows for the swapping out of various new model components and therefore facilitates community development and model inter-comparison. Third, via its placement on a public GitHub repository under a permissive open-source license (the MIT license), Mimi-PAGE is available for inspection, use, and further development by all researchers. Fourth, our consistent programming conventions, along with how-to-read and how-to-run guides, are documented in the public GitHub repository.

The flexibility now enabled by modular IAMs such as Mimi-PAGE was recommended by the 2017 National Academy of Sciences committee reviewing estimation of the SCC^8^. Modularity increases functionality for a range of users such that specific pieces of a given model can receive targeted development from the relevant scientific community. In addition, the NAS panel recommended model code be available for “review, use, and modification by researchers”, something supported by the open-source implementation that we describe here.

### Modular Approach to Modeling and Mimi

Integrated assessment models draw on a wide range of academic disciplines for their equations and calibrations of key parameters. Ideally one would like to have input from scientists in these different disciplines in the construction, review and discussion of integrated assessment models. In practice this is often difficult because most integrated assessment models appear to the outside world like one big black box model and it can be very difficult to work on say just one specific aspect of an IAM (e.g. the carbon cycle). In the past, these difficulties have sometimes discouraged domain experts to get involved with IAMs.

A modular approach to integrated assessment models attempts to alleviate these issues by splitting an IAM into different components. It then uses a computational platform where these individual components can be easily identified, used and investigated in isolation and also easily be replaced by alternative scientific formulations. The integrated assessment model is then a container of coupled individual model components, an approach recommended by the recent National Academy of Science report on the Social Cost of Carbon Dioxide^8^.

We use the Julia Mimi.jl package to achieve these goals. Mimi allows us to split a large model like PAGE09 into a number of smaller components, that each correspond to a well defined area of the problem domain, that in many cases has a clear disciplinary link. Each component can be tested and used individually, and then easily be combined with the other components to form the complete PAGE09 model. Mimi.jl has been used previously for other models, for example the FUND model has adopted it as its platform, and a recent publication used a port of the RICE model to the Mimi platform^9,10^.

### The Policy Analysis of the Greenhouse Effect (PAGE) Model

Mimi-PAGE is an open-source recreation of the PAGE09 model, the latest iteration of the PAGE model, originally developed in 1993 (refs^7,11,12^). PAGE09 is an eight-region model that takes income, population and emissions policy as inputs, and models the effect of emissions of four different greenhouse gases - carbon dioxide, methane, nitrous oxide, and a collection of other greenhouse gases (termed ‘linear gases’ in PAGE because of the linear relationship between concentration and radiative forcing).

PAGE09 uses a radiative balance climate model to simulate global and regional temperature changes and sea-level rise. Warming and sea-level rise cause damage in 4 different sectors: market sectors (e.g. agriculture, forestry, tourism etc), non-market sectors (e.g. mortality and ecosystem damages), sea-level rise (i.e. coastal flooding) and a stochastic discontinuity (a one-time tipping point resulting in a large loss of GDP). PAGE also models an exogenously-determined adaptation policy that reduces impacts in three of the sectors for a price. Finally, the costs of reducing emissions below their business-as-usual path (called preventative costs in PAGE) are also calculated. PAGE09 simulates outcomes at variable time-steps starting in the default model at 2008 and ending in 2200.

**Figure 1:**
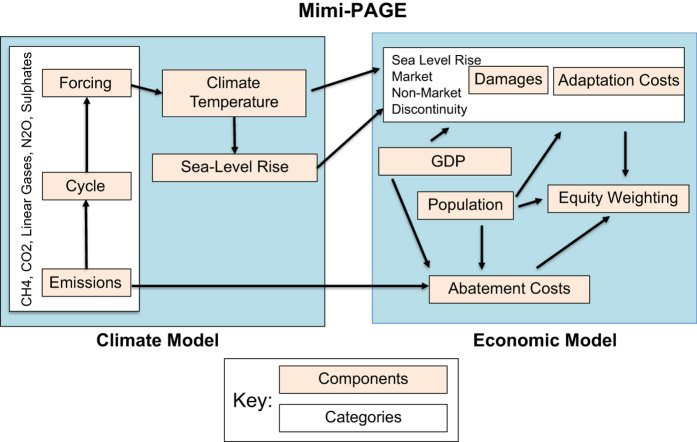
Schematic diagram of the component structure of Mimi-PAGE. For visual simplicity, where a certain type of component is replicated multiple times (e.g. CH4, CO2, Linear Gases, N2O, and Sulphates each have an emissions component), we have shown it only once as a “category” and identified the multiple instances that it appears in the model. The full set of components with exact names included in Mimi-PAGE is given in Box 1 (Methods).

A key feature of PAGE09 is the inclusion of uncertainty distributions (triangular distributions in the default version of the model) for over 100 key scientific and economic parameters. This allows PAGE to be run probabilistically through a Monte Carlo procedure to generate outcome distributions. More details on PAGE09 are given in Hope (2013)^7^. Figure 1 gives a diagram showing the components of PAGE09 as implemented in Mimi-PAGE.

## Results

### Validation Exercises

Model output was validated by comparison with output from PAGE09 run in its native Excel using the @RISK plug-in, both for a deterministic run with mean values for the inputs, and for the full probabilistic implementation. Additional details on component and model testing is given in the Methods section. Both PAGE09 and Mimi-PAGE can run any user-defined emissions scenarios, but validation results presented here focus on the business-as-usual emissions scenario (Policy A in PAGE09). Mimi-PAGE was also tested against a mitigation policy scenario (Policy B in PAGE09), with results similar to those presented here for the business-as-usual scenario. Tests of the Policy B emissions scenario are available for inspection in the GitHub repository (see Code Availability).

**Figure 2:**
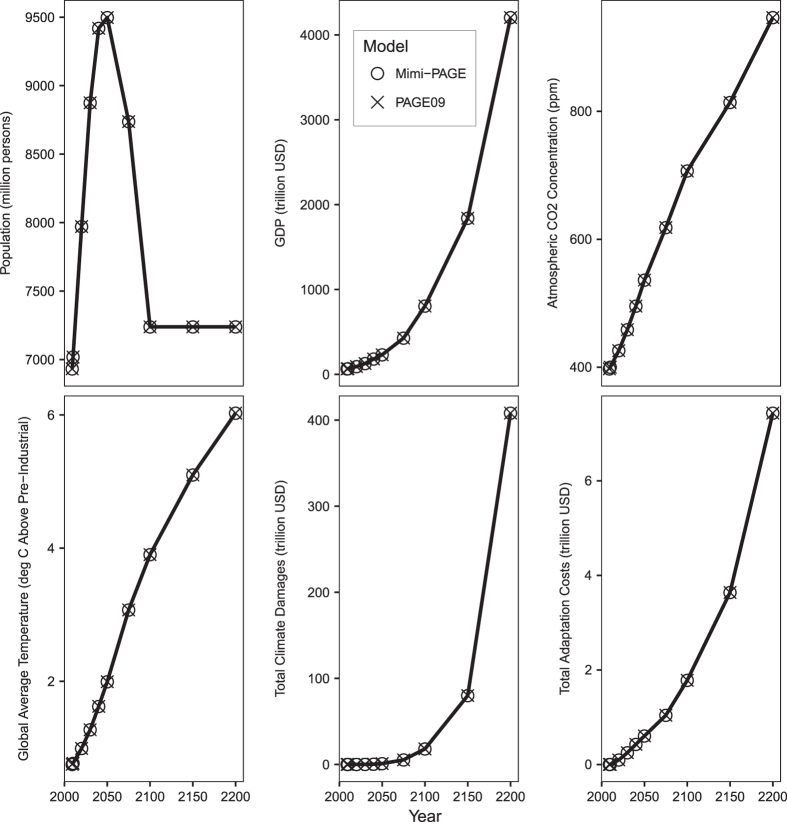


Figure 2 shows the evolution of key variables in the business-as-usual scenario for both PAGE09 and Mimi-PAGE under the deterministic run of the model (using mean parameter estimates). It shows the very close match between the two model implementations for both socio-economic variables (population and GDP, which are calculated within the model from specified baseline values and growth rates), climate variables (CO_2_ concentration and global temperature) and economic results (climate impacts and adaptation costs). The final model output ‘Total Effect’ variable (the discounted costs of climate damages, adaptation, and prevention for all regions and time periods), which depends on results from all model components, agrees to within 0.003%. The match for other example variables is even closer, as documented in Table 1. To the best we can determine, the differences documented in Table 1 result from the use of finite precision floating point arithmetic, a standard approach in almost all computational work.

**Figure 3:**
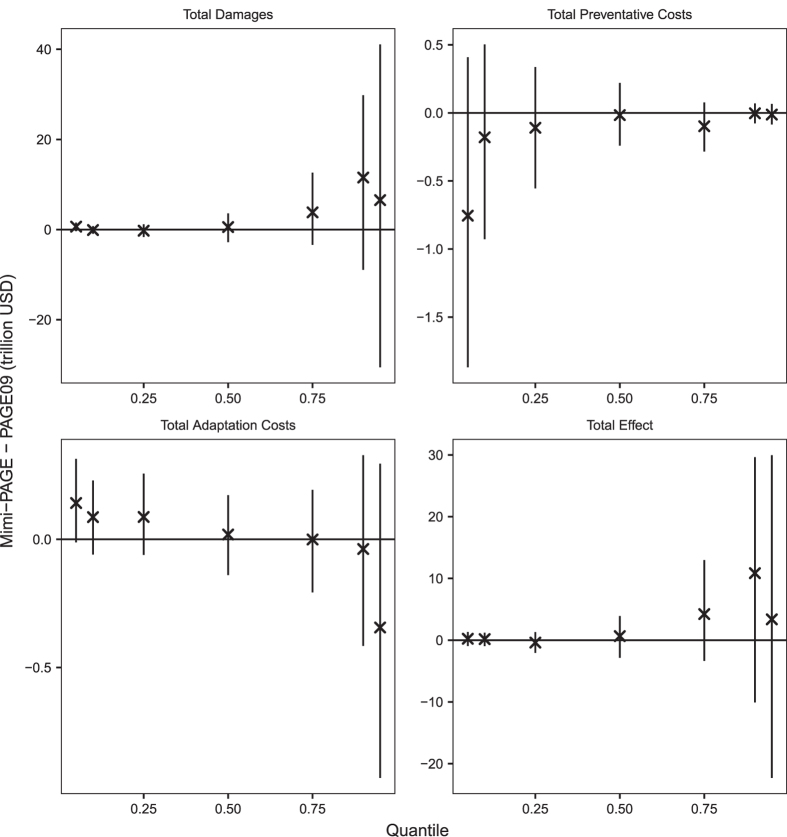
Comparison of quantiles from the distribution of 4 endpoint variables obtained from 100,000 Monte Carlo runs of Mimi-PAGE and PAGE09. Error bars show the 95% confidence interval associated with sampling uncertainty of the Monte Carlo procedure.

For the full probabilistic version of the model we validated 7 quantiles (0.05, 0.1, 0.25, 0.5, 0.75, 0.9 and 0.95) of the distribution of 4 model endpoint variables – total discounted damages, total discounted preventative costs, total discounted adaptation costs, and total effect (described above). Distributions of all four variables are based on 100,000 Monte Carlo simulations of both Mimi-PAGE and PAGE09. Figure 3 shows a comparison between the quantiles of the distributions with 95% confidence intervals based on the sampling uncertainty associated with Monte Carlo sampling^13^. Sampling uncertainty is introduced in the validation of Monte Carlo procedures because input parameters are random draws meaning output distributions would be expected to converge only asymptotically. Even with 100,000 draws, sampling uncertainty might be expected to be significant, particularly in the tails of the distributions. Figure 3 suggests that any difference between the Mimi-PAGE and PAGE09 is well within sampling error (i.e. all confidence intervals include zero).

### Speed comparison

One major benefit of the Julia language is its improved computational speed over many other programming languages^14^. In this context it means that Monte Carlo simulations can be done faster in Mimi-PAGE compared to PAGE09. Table 2 provides a comparison of processing times for 1,000 and 10,000 Monte-Carlo simulations run in Julia (Mimi-PAGE) and in Excel (PAGE09) using the @RISK plugin. All tests were performed on the same computer (an Intel Core i7-4770 @3.4 GHz CPU with 16GB of main memory).

Table 2 shows a substantial speed advantage of the Julia implementation. Using only a single core, the Mimi-PAGE version is 11.9 times faster than PAGE09 for both one and ten thousand Monte-Carlo runs. The @RISK plugin does support multi-threading, something not currently possible with Mimi-PAGE. However, even the multi-threaded runs of PAGE09 on this quad-core processor ran more than three times slower than the equivalent single-threaded runs in Julia.

## Discussion

The results presented above demonstrate the faithful replication of PAGE09, both of the single run using central parameter estimates and of the full Monte Carlo distribution, in Mimi-PAGE. Benefits of this new implementation of the model include substantially-reduced processing time and the use of free, open-source software that increases accessibility of the PAGE09 model to researchers and policy-makers. The component-based implementation of Mimi-PAGE is designed to enable future community development and improvement, as well as easier inter-comparison with other IAMs.

The Mimi project is still on-going and does not as yet support all the functionality of PAGE09. As noted above, Mimi-PAGE cannot yet be run using multiple-threading. In addition, PAGE09 can be run in an optimization mode (see for example Hope^15^). While there are good optimization packages available in Julia, there is not yet a built-in optimization capability in Mimi-PAGE. Future work on the Mimi platform and on the Mimi-PAGE model will introduce these features.

The recoding of PAGE09 by an independent team in a new platform is also itself an important exercise in model validation and replication. Nordhaus^16^ has pointed out the potential for errors or bugs in complex computational models and notes that independent recoding of the model from scratch can be a valuable confirmation of the original model. We note that we were able to recreate PAGE09 almost entirely from the thorough documentation of model structure already published, using the original model only for validation and testing of a working version of Mimi-PAGE.

## Methods

### Overview of Mimi-PAGE

The Mimi.jl coding platform allows for modular model development by dividing models into constituent components. Figure 1 gives a schematic of the component structure developed for Mimi-PAGE. In all there are 32 components, listed specifically in Box 1. Each component is initialized with specific parameter values or exogenous input data and is connected to other components through shared variables (i.e. variables calculated in one component are used as input into another component). Each component calculates a single time-step at a time, and the components are executed in an order so that each depends only on components excuted before it.

Mimi-PAGE also supports the Monte Carlo functionality of PAGE09. Components containing one or more of the more than 100 random variables can be initialized with random draws from the uncertain parameter distributions. This can be iterated multiple times in a Monte Carlo procedure in order to simulate distributions of outcome variables. The default distributions are triangular distributions, but these could be modified by the user if desired to any of the 42 continuous univariate distributions supported by the ‘Distributions.jl’ package in Julia, or to one of the multivariate distributions provided there.

Mimi-PAGE was coded using documentation provided in Hope^12^ and Hope^7^ using best coding practices. Model equations were coded directly from the model documentation, without reference to the PAGE09 model, which was only used for validation and testing of a working version of Mimi-PAGE. Each component underwent a scientific review (to check consistency with model documentation) and a technical review (to catch code errors and bugs) by two different people before being merged into the master code version. A full suite of automated validation tests were developed to compare the output of individual components and the full model against values from PAGE09 (additional details in Results and Products Provided sections). These tests are automatically run for new commits to the code repository and will produce errors if changes to the model result in failure of the validation tests. Other best coding practices include full documentation (described in detail below), use of version control via git, use of an open-source language, and consistent conventions^6^. In addition, the data and repository for the replication of this paper have been made available (see Code Availability).

Mimi-PAGE requires the Julia language to run as well as the open-source Mimi package. The file “main_model.jl” in the source folder, “src”, includes code for a single run of Mimi-PAGE using the means of the parameter distributions. The file “montecarlo.jl” includes code for running the full Monte Carlo model. The number of iterations as well as the variables output from the Monte Carlo simulations can be set by the user here. Additional information on using Mimi-PAGE can be found in the “Getting Started” and “Technical User Guide” sections of the model documentation.

### Code Availability

Code for Mimi-PAGE, as well as input data, validation tests, and documentation are available in a public Github repository at https://github.com/anthofflab/mimi-page-2009.jl^17^. Code to reproduce the validation figures and tables included in this paper are available at https://github.com/anthofflab/paper-2018-mimi-page-replication^18^.

### Products Provided

This section describes the work products released to the community as part of the Mimi-PAGE repository, as organized within the model source code repository.

#### Model Source Code

The source folder (“src”) contains all equations making up the PAGE09 model, divided into core model components as described in the previous section, contained in the “components” sub directory. These were implemented in Julia based on documentation of the PAGE09 model provided in Hope^7^ and Hope^12^. The code is designed to easily connect the Julia equations to those provided in the original documentation. Comments in the code refer back to page or equation numbers in the documentation. Variable naming convention retains the variable names from PAGE09 with an additional, longer description. For example, the variable ‘rtl_g_landtemperature’ retains the PAGE09 naming convention of referring to global land temperature as rtl_g while adding the additional descriptor “landtemperature”. The source code folder also contains scripts to run the model either using just the mean of the uncertainty distributions or in full Monte Carlo mode, or to run only the climate portion of the model.

#### Tests

The test folder (“test”) contains code that tests each individual component and the full model against output from the standard PAGE09 model version. These tests ensure the Mimi-PAGE replication is faithfully reproducing the original version. Each component has an associated test file in the test folder, with the naming convention ‘test_*<component>*.jl’.

In each test, components are initialized using data from the original default PAGE09 model and then their output variables are compared to the same variables from PAGE09. For example, testing the climate system component requires initializing it using radiative forcing from the original model and then comparing calculated warming against warming from PAGE09. Tolerance of individual tests can be set by the user as input to the @test Julia function. The entire model can be tested by running ‘test_mainmodel.jl’ which compares output of the full Mimi-PAGE model against PAGE09 for a business-as-usual emissions scenario. Tests are also provided for a mitigation policy scenario ‘test_mainmodel_policyb.jl’ and for the stochastic Monte Carlo runs of the model (‘test_montecarlo.jl’).

#### Model Data and Parameters

Input data used to initialize the PAGE model is included in the data folder (“data”) as .csv files. These are original data from the default PAGE09 model that were extracted from the Excel version using a script and then saved as .csv files by the author team. Key input data include population, income, and business-as-usual emissions growth rates for each of the 8 regions over the model time period (2008-2200). An alternative emissions scenario is also included in the ‘policy-b’ folder.

The data folder also contains model parameters used to initialize PAGE that vary by region or time-period as .csv files. In particular, the large number of region- and sector-specific variables describing the exogenously set adaptation policies are included here. Scalar parameters are directly included in the relevant component source files, in the “add<*component>*” function. PAGE09 includes distributions that capture uncertainty in over 100 key parameters allowing the model to be run in Monte Carlo mode. For components that include uncertain parameters, the parameters of the triangular distribution are given in the “randomize<*component>*” function within the relevant component file.

Data on endogenous variables calculated within PAGE09 are also supplied as part of the component testing functionality. The “validationdata” folder, within the “test” folder, contains output of key variables from the original PAGE09 model that are used to initialize individual components or to test their results (see example in previous section). Key variables used for testing include emissions and atmospheric concentration of greenhouse gases, radiative forcing, temperature change, sea-level rise, climate damages, and abatement and adaptation costs.

#### Documentation

The Mimi PAGE repository also includes substantial scientific and technical documentation in the “docs/src” folder, complementing documentation of PAGE09 in Hope^12^. Sections include documentation on how to get started running Mimi–PAGE in Julia (including required Julia packages), description of the Mimi model components and validation of the model against the original PAGE09 output, and technical documentation on the Mimi component-based modeling environment and coding conventions.

## Additional information

**How to cite this article**: Moore, F. C. *et al*. Mimi-PAGE, an open-source implementation of the PAGE09 integrated assessment model. *Sci. Data* 5:180187 doi: 10.1038/sdata.2018.187 (2018).

**Publisher’s note**: Springer Nature remains neutral with regard to jurisdictional claims in published maps and institutional affiliations.

## Figures and Tables

**Table 1:** Numerical differences in example model variables for deterministic runs of Mimi-PAGE and PAGE09 under the business-as-usual emissions scenario.

**Model Variable**	**% Difference (Mimi-PAGE vs PAGE09)**
Global Temperature in 2200	0.001
Global GDP in 2200	1.39e-8
Global Preventative Costs in 2200	1.45e-6
Global Adaptation Costs in 2200	5.61e-7
US Per-Capita Consumption after All Damages in 2200	0.0003
Total Effect	0.003

**Table 2:** Speed comparisons of Monte Carlo runs of Mimi-PAGE run in Julia and PAGE09 run in Excel using the @RISK plugin.

**Model**	**Number of Runs**	**Multi-Threading?**	**Time (seconds)**
Mimi-PAGE	1,000	FALSE	8
PAGE09	1,000	FALSE	95
PAGE09	1,000	TRUE	31
Mimi-PAGE	10,000	FALSE	79
PAGE09	10,000	FALSE	937
PAGE09	10,000	TRUE	260

## Details on Mimi-PAGE Structure and Components

*Default Time Periods*: 2008 (initial baseline), 2009, 2010, 2020, 2030, 2040, 2050, 2075, 2100, 2150, 2200.

*Default Geographic Regions*: Europe (EU), the United States (US), other countries in the Organization for Economic Cooperation and Development (OECD), the former Soviet Union (USSR), China, South-East Asia (SEAsia), Africa, and Latin America (LatAmerica)

*Components:*

1. Climate Components: CO2emissions, CO2cycle, CO2forcing, CH4emissions, CH4cycle, CH4forcing, N2Oemissions, N2Ocycle, N2Oforcing, lgemissions, lgcycle, lgforcing, totalforcing, sulphateforcing, climatetemperature, sea-level-rise
2. Socio-Economic Components: population, gdp
3. Damage Components: slrdamages, marketdamages, nonmarketdamages, discontiuity
4. Policy Cost Components: abatementcosts_CO2, abatementcosts_CH4, abatementcosts_N2O, abatementcosts_Lin, totalabatementcosts, adaptationcosts_sealevel, adaptationcosts_economic, adaptationcosts_noneconomic, totaladaptationcosts
5. Discounting and Equity-Weighting Components: equityweighting
